# Evaluation of different strains of *Saccharomyces cerevisiae* for ethanol production from high-amylopectin BRS AG rice (*Oryza sativa* L.)

**DOI:** 10.1038/s41598-022-06245-0

**Published:** 2022-02-08

**Authors:** Isabela C. Almeida, Thályta F. Pacheco, Fabricio Machado, Sílvia B. Gonçalves

**Affiliations:** 1 Instituto de Química, Universidade de Brasília, Campus Universitário Darcy Ribeiro, Brasília, DF CEP: 70910-900 Brazil; 2 Embrapa Agroenergia, Parque Estação Biológica, PqEB s/n°, W3 Norte, Brasília, DF CEP: 70770-901 Brazil; 3 Present Address: School of Chemistry, University of Nottingham, University Park, Nottingham, NG7 2RD UK; 4 Present Address: Faculty of Engineering, University of Nottingham, University Park, Nottingham, NG7 2RD UK

**Keywords:** Biotechnology, Chemical engineering

## Abstract

Ethanol is the main biofuel produced by fermentation route and the search for new feedstocks to produce fuel ethanol is still a great challenge. This work aims to compare the ethanol production from a new irrigated rice cultivar BRS AG to the conventional cultivar BRS PAMPA applied in Brazil. Six different commercial strains of *Saccharomyces cerevisiae* (BG-1, CAT-1, FT-858, JP-1, PE-2, and SA-1) were applied in fermentation reactions. Fermentations performed with BRS PAMPA rice revealed that the highest yields were achieved with strain SA-1, corresponding to 93.0% of the theoretical maximum and final ethanol concentration of 58.92 g L^−1^, and with CAT-1, a yield of 92.7% and final ethanol concentration of 58.93 g L^−1^. For the fermentations with BRS AG rice, the highest yields were obtained with strain FT-858, exhibiting a 89.6% yield and final ethanol concentration of 62.45 g L^−1^, and with CAT-1, 87.9% yield and final ethanol concentration of 61.25 g L^−1^ were achieved. The most appropriate microorganism for ethanol production using BRS PAMPA rice and BRS AG rice was CAT-1. Comparatively, the ethanol yield and productivity using BRS AG were higher than those observed for BRS PAMPA for all strains, except for PE-2 and SA-1 that led to very similar results. The experimental results showed that the giant rice BRS AG is an excellent feedstock for fuel ethanol production in lowland fields.

## Introduction

The search for renewable and clean alternative fuels that can supply the global energy demands has increased in recent years. That is because burning fossil fuels, as oil, coal and natural gas emit greenhouse gases, which are the major contributors to the global temperature rise^1^. It is signposted that by 2050, a minimum 40% reduction in greenhouse gases emissions is obligatory to sustain the average increase < 1.5 °C^2^. In addition to environmental problems, fossil fuels may deplete^1^.

In this way, ethanol has been considered as the most viable and potential alternative to reduce the use of fossil resources and its production has been increasing over the years^3^. In 2019, the global industrial demand for ethanol was 116.9 billion liters, and it is projected to grow at a compound annual growth rate of 2.5% to reach 135.5 billion liters by 2025^4^.

The industrial production of ethanol consists of fermentation processes, using microorganisms to convert sugars into alcohol. The sugary feedstocks are those that contain simple sugars as monosaccharides (glucose or fructose) or disaccharides (sucrose or saccharose), which can be fermented using a potential microbial strain. The *Saccharomyces cerevisiae* strain is most used for ethanol fermentation^4^. Furthermore, many biomasses can be used as a source of sugar in the fermentation process^5^. Currently, ethanol is mainly produced from food-based crops such as corn and sugarcane^3^. In the fermentations using starch as a source of carbon, an initial step is necessary to promote starch hydrolyze into monomeric sugars before fermentation to allow ethanol production.

Currently, the United States of America and Brazil are the major producers of bioethanol. They used food crops as feedstock via a biochemical route^4^. The United States of America uses starch of corn as a source of carbohydrate in the fermentation process^6^. On the other hand, Brazil uses sucrose present in sugarcane juice to obtain ethanol^7,8^. In 2019, they produced about 15.8 and 8.6 billion gallons of ethanol, which contributes about 89% of the world’s total ethanol production^4^.

High yields of ethanol production are obtained by using starch as raw material^9^. Starch crops such as corn, barley, wheat, potatoes, sweet potatoes, cassava and rice are promising for ethanol production due to their economic viability and availability. However, using these substrates for ethanol production will cause serious threats to food supply and arable lands^3^. As a result, the prices of food products can be affected^10^.

In this context of the productive chain and the search for renewable energies that are economically viable and that do not compete with the food industry, Embrapa Clima Temperado, that is a Brazilian Agricultural Research Corporation developed an irrigated rice cultivar, BRS AG. This cultivar was developed to be used as a raw material to produce cereal alcohol or animal feed^11,12^.

The BRS AG is the result of a simple cross between the American irrigated rice cultivar Gulfmont and the SLG1lineage (super large grain), of Japanese origin^11^. The dimension of BRS AG grains is larger than those of conventional rice, one thousand grains of this rice have an average weight of 52 g, while the conventional rice cultivars show lower values, such as 25.6 g for BRS Pampa^13^. Added to that, the average yield of BRS AG planted is approximately 8.2 ton·ha^−1^, approaching the average yield obtained by the BRS Pampa 9.2 ton·ha^−1^^11^.

The plants of this cultivar have a biological cycle of around 126 days, from emergence to maturity. The average plant height is 110 cm, and the thickness of the stem is 5.5 mm, which gives them strong culms, resistance to lodging, despite this high stature of the plants^11^. In contrast, the plants of BRS Pampa cultivar have a precocious biological cycle of around 118 days, from emergence to maturity and the average plant height is 96 cm^11^.

The grains of BRS AG are extremely floury, and their productivity exceeds 12 tons per hectare, which corresponds to almost double the traditional cultivars. The BRS AG rice has a low amylose-amylopectin proportion, which is responsible for inferior quality in cooking. In addition, this rice has very large grains, which does not meet the standards of Brazilian industry and consumption. In addition to high productivity, and not being used in human food, its grains have a large amount of starch, making it extremely attractive for ethanol production^11,14^.

The BRS AG, as an irrigated rice cultivar, can be regarded as an attractive raw material naturally produced in lowland fields, characterized by presenting hydromorphic, poorly drained soils, which imposes strongly restriction on other cultures such as soybean, maize and sorghum, among others^11^.

One ton of the BRS AG grain is capable of producing almost 430 L of ethanol, a high value compared to other raw materials also used in the fermentative process to produce ethanol^14^. For example, it is necessary one ton of sugar cane to produce 90 L of ethanol, and the productivity is 60–120 ton·ha^−1^. When corn is used as raw material to ethanol production, the yield is 370–460 L·ton^−1^ and the productivity is 7.5–10.0 ton·ha^−1^. When conventional rice is used as biomass for ethanol production, the yield is 420–450 L·ton^−1^ and the productivity is 7.5 ton·ha^−1^^15^. Thus, it is possible to see that a higher yield in ethanol production is obtained when starchy biomass is used. However, when the raw material to the alcoholic fermentation is amylaceous, it is necessary to hydrolyze the starch into monomeric sugars before the fermentation process, decreasing the productivity. This is because *S. cerevisiae* strain does not convert the starch to ethanol directly^16^.

Starch-based feedstocks are comprised of storage polysaccharides in the form of starch, which in turn is formed of two chains of glucose, straight-chained amylose (α-1,4) and branched amylopectin (α-1,4; branching at α-1,6). The conversion of this complex polysaccharide requires hydrolysis of starch into fermentable sugars with two steps. The first step is liquefaction using α-amylase at high temperature, which hydrolyzes the α-1,4 linkage of starch causing the release of maltose, dextrin, maltopentoses, and maltotriose. The second step is complete hydrolysis using amyloglucosidase (AMG), which breaks down the α-1,4 as well as α-1,6 linkages of starch resulting in the release of glucose along with other free soluble sugars if any, followed by the fermentation^4^.

In order to lead to a higher final concentration of ethanol and higher yield, the fermentation process depends on factors such as temperature, pH, sugar concentration, fermentation time and agitation rate^17^. The optimal temperature of *S. cerevisiae* during the fermentation process is around 26 °C to 35 °C. High temperatures may lead to bacterial contamination, which may result in flocculation, yeast cell viability reduction, and a decrease in the yield process. Bacterial contamination with more than 107 colony-forming units per mL may result in a reduction of 1% to 5% in final yield^18^. Choosing the most appropriate strain for the process is important to successfully obtain an alcoholic fermentation. The better yeasts present good fermentative efficiency, high fermentation speed, conversion efficiency, resistance to ethanol, resistance to low pH, resistance to antiseptics and genetic stability^19^.

The raw material used in Brazil in alcohol industries is sugarcane^8^, and the main yeast used in distilleries are CAT-1, PE-2, BG-1, SA-1, FT858^19,20^. These strains have been used in distilleries that account for 70% of all ethanol production in Brazil^16^. In this way, according to the reaction conditions that will be adopted in the fermentation, a given strain may be more suitable than another. For example, CAT-1 is tolerant to the aluminum present in the sugar cane juice and, because of this, is one of the most used yeasts in the Brazilian process^21^.

High alcohol concentrations in the fermentative medium make it difficult for the *S. cerevisiae* cells to grow up^7^. The strain PE-2 is also widely used in Brazil in the fermentation processes because this strain shows tolerance to alcoholic contents of up to 19% (v/v)^22^.

In general, industrial strains exhibit resistance to low pH, such as BG-1 and SA-1. In addition to being resistant to low pH, FT-858 also produces low foaming during fermentation and is tolerant to high alcohol contents^21^. On the other hand, the strain JP-1 has tolerance to high temperatures and does not flocculate^23^.

This work presents an experimental study on the ethanol production using both BRS PAMPA rice (fine-grained rice cultivar used commercially) and BRS AG rice (N.B. rice cultivar developed by Embrapa Clima Temperado) along with different commercial strains of *S. cerevisiae*, such as BG-1; CAT-1; FT-858; JP-1; PE-2; SA-1. The best microorganism in the fermentation process of each rice cultivar was evaluated based on the values obtained from yield, ethanol concentration and ethanol productivity. In this way, a step of hydrolysis of the starch present in BRS PAMPA rice and BRS AG rice was performed, followed by fermentation with different strains of *S. cerevisiae*. High-performance liquid chromatography (HPLC) analysis determined the final ethanol concentration and the concentration of fermentation co-product, glycerol. The yield and productivity obtained were compared for the two rice cultivars.

## Materials and methods

### Materials

BRS PAMPA rice was supplied by JOSAPAR (Joaquim Oliveira S.A, Rio Grande do Sul, Brazil) and BRS AG rice, developed with exclusive purposes for ethanol production, was kindly donated by Embrapa Clima Temperado (Pelotas, RS, Brazil). Both BRS PAMPA and BRS AG rice samples were received without hulls.

### Preparation of BRS PAMPA rice and BRS AG rice

Samples of BRS PAMPA rice and rice of cultivar BRS AG were milled on a knife mill type Willey Fortinox Star FT-60 to an average particle size of 696.8 ± 25.4 μm.

### Enzymatic hydrolysis

#### Study of enzyme hydrolysis parameters

The hydrolysis reactions of starch present in BRS PAMPA rice were carried out in 0.1 M sodium citrate buffer solution with pH 5.5, and in an Eppendorf Fermentation System, model Dasgip Bioblock. In order to prepare 0.1 M sodium citrate buffer solution, at pH 5.5, we used: citric acid (grade P.A.); sodium citrate dihydrate (grade P.A.); and distilled water.

The hydrolysis of the starch present in the rice samples was performed in two steps. The first step was the starch liquefaction, done by using the enzyme α-amylase Termamyl 2X (Novozymes, batch AYVD0061, enzymatic activity: 240 KNU-T/g), followed by the saccharification using the enzyme amyloglucosidase (AMG 300 L, Novozymes, batch AMN06024, enzymatic activity: 300 AGU/mL), to obtain the glucose.

The ground rice and 0.1 M sodium citrate buffer solution, pH 5.5, were added to a pre-weighed beaker, considering the glucose concentration of 120 g L^−1^. The glucose concentration was determined by high-performance liquid chromatography (HPLC) (as described in “High-performance liquid chromatography (HPLC)”).

The mixture was transferred to the reactor Eppendorf Fermentation System, model Dasgip Bioblock. The system was heated to 90 °C under 300 rpm stirring. Upon reaching the temperature, the Termamyl 2X enzyme was added to the reactor, 10 times more than the dosage recommended by the manufacturer Novozyme.

A preliminary study developed by our research group evaluated the amount of enzymes that would lead to suitable hydrolysis of starch to glucose, showing that the required concentration of enzymes must be increased in order to guarantee a total conversion of the hydrolysis process. As the main objective of this study is evaluating fermentation reactions with different commercial lines of *S. cerevisiae* the excess of the enzyme during hydrolysis does not hinder the process. Thus, the enzyme was added 10 times in excess of the amount recommended by the manufacturer Novozyme.

To determine the best condition for the hydrolysis process, the reaction time of α-amylase in the starch liquefying step was varied. A hydrolysis reaction without this enzyme was carried out, as well as hydrolysis in which the enzyme Termamyl 2X acted for 30 min, 1 h, 2 h and 3 h.

After the liquefaction step, 1 M citric acid solution was added to the mixture in order to reduce the pH to 4.5, and the system was cooled to 60 °C. These are the optimal conditions of amyloglucosidase action. The enzyme AMG 300 L was also added 10 times in excess of the dosage recommended by the manufacturer Novozyme. The saccharification reaction was carried out for 3 h. Although the commercial enzyme extract has high amounts of sugar, the enzyme extract was diluted ten times for hydrolysis. Thus, it was strongly expected that the amount of sugar present in the enzymatic extract used in the starch hydrolysis step would not be sufficient to interfere with fermentation reactions and ethanol production.

The best reaction condition was determined considering the final glucose amount, as quantified by HPLC (described in “High-performance liquid chromatography (HPLC)”).

#### Hydrolysis of starch present in BRS PAMPA rice and BRS AG rice for later fermentation reactions

The enzymatic hydrolysis reactions of starch present in rice samples were performed in buffered medium, sodium citrate buffer solution with pH 5.5. The ground rice and 0.1 M sodium citrate buffer solution, pH 5.5, were added to a beaker, considering the sugar concentration of 120 g L^−1^. A system with a heating plate, temperature controller and mechanical stirrer was set up. The beaker with the rice solution and sodium citrate buffer was placed on a heating plate.

The mixture was heated to 90 °C under stirring. At this temperature, the enzyme Termamyl 2X was added in excess, to act on the starch liquefaction. After one hour, time of this alpha-amylase action, the pH of the mixtures was adjusted to 4.5 with 1 M citric acid solution addition. The temperature was lowered to 60 °C, as these are the optimal conditions of amyloglucosidase action^24^. The enzyme AMG 300 L was added in excess to the system and the saccharification reaction went for further 3 h.

### Yeast cell growth

The culture medium for cell growth of *S. cerevisiae* strains were prepared using bacteriological peptone, yeast extract powder, D ( +) anhydrous glucose, grade P.A., agar, and distilled water. To obtain cell mass, we used medium YPG 10% as a cultivation, containing 1.0% (w/v) yeast extract, 2.0% (w/v) bacteriological peptone, 2.0% (w/v) glucose. Firstly, the glucose was solubilized in distilled water, followed by the addition of yeast extract and peptone. The mixture was sterilized in an autoclave at 121 °C, 1 atm for 30 min. The flasks containing cells of different *S. cerevisiae* commercial strains were incubated for 16 h in an orbital shaker stirring system (MAXQ5000, Thermo Scientific) at 32 °C, under the agitation of 150 rpm. Six commercial ethanol production strains of *S. cerevisiae*, coded as BG-1; CAT-1; FT-858; JP-1; PE-2; SA-1, were used in this study as inoculum. These strains derive from commercial lines of S. cerevisiae used in industrial processes of ethanol production and are strains isolated from fermentation processes by Fermentec Ltda. (Piracicaba, São Paulo, Brazil).

### Fermentation reactions

The fermentation reactions of glucose from BRS PAMPA and BRS AG rice (only) were performed in an orbital shaker (MAXQ5000, Thermo Scientific), at 32 °C and 150 rpm, using 250 mL The Erlenmeyer flasks were closed with a cotton plug and only the hydrolysate material was added to the fermentation system. The fermentations were carried out in triplicate for each strain of *S. cerevisiae.* The hydrolyzed was added so the initial glucose concentration of 120 g L^−1^. For the inoculum, 10% of yeast was used in relation to this glucose concentration. The volumes of the Erlenmeyer flasks were 250 mL, and the hydrolyzed added in each Erlenmeyer was 140 mL, and the fermentations were carried out for 48 h.

### High-Performance liquid chromatography (HPLC)

The quantification of the glucose after de hydrolysis, as well as the glucose and ethanol during fermentations, was done through high-performance liquid chromatography (HPLC) equipment carried out on the Infinity 1260 Agilent Technology with an Aminex HPX-87H chromatographic column (BioRad), using a 0.005 M sulfuric acid solution as mobile phase made up with 97.8% pure sulfuric acid, and purified distilled water in a Milli-Q system. For each use of HPLC equipment, 2 L of sulfuric acid solution were prepared as mobile phase. The solution was degassed in an ultrasonic bath for 15 min and added to the HPLC system.

### Evaluation of the fermentation performance

After the fermentation reactions of the hydrolysates of BRS PAMPA rice and BRS AG rice, fermentation parameters were calculated, as: substrate to product conversion factor, yield, productivity, and substrate consumption speed.

The conversion factor ($Y_{{{P \mathord{\left/ {\vphantom {P S}} \right. \kern-\nulldelimiterspace} S}}}$) of the substrate in the product was expressed in g_ethanol_/g_glucose_. The calculation was performed according to Eq. (1):

1 $$Y_{{{P \mathord{\left/ {\vphantom {P S}} \right. \kern-\nulldelimiterspace} S}}} = - \frac{dP}{{dS}} = - \frac{{P_{f} - P_{i} }}{{S_{f} - S_{i} }}$$

where *P* is the product concentration (g L^−1^). *P*_*f*_ and *P*_*i*_ are the final and initial product concentrations, respectively, and *S* is the substrate concentration (g L^−1^). *S*_*f*_ and *S*_*i*_ are the final and initial substrate concentrations, respectively.

The fermentation yields (*R*) of BRS PAMPA rice and BRS AG rice hydrolysates were obtained based on the theoretical yield from the Gay-Lussac equation, where 51.1 g of ethanol per 100 g of glucose. Thus, Eq. (2) was used to determine the fermentation yields in relation to the theoretical maximum (0.511 g ethanol per g of consumed glucose)^25^:

2 $$R = {{\left( {100 \cdot Y_{{{P \mathord{\left/ {\vphantom {P S}} \right. \kern-\nulldelimiterspace} S}}} } \right)} \mathord{\left/ {\vphantom {{\left( {100 \cdot Y_{{{P \mathord{\left/ {\vphantom {P S}} \right. \kern-\nulldelimiterspace} S}}} } \right)} {0.511}}} \right. \kern-\nulldelimiterspace} {0.511}}$$

The productivity of the process ($\wp$) is expressed in g_ethanol_ L^−1^ h^−1^ (Eq. 3). Where, *t* is the fermentation time (h), and *t*_*f*_ and *t*_*i*_ are the final and initial fermentation time, respectively.

3 $$\wp = \frac{dP}{{dt}} = \frac{{P_{f} - P_{i} }}{{t_{f} - t_{i} }} = \frac{{\text{Produced ethanol}}}{{\text{Fermentation time}}}$$

The rate of substrate consumption (V) was determined by the amount of glucose consumed (g L^−1^) in relation to the fermentation time (h). The calculation was performed according to Eq. (4):

4 $$V = \frac{dS}{{dt}} = \frac{{S_{i} - S_{f} }}{{t_{f} - t_{i} }}$$

where S is the substrate concentration (g L^−1^) and t is the fermentation time (h).

### Statistical analysis

Statistical analysis was performed using the TIBCO Statistica® 13.3.0. and OriginPro 2021 9.8.0.200 with a significance level (p) of 0.05. The parametric data analysis was carried out using analysis of variance (ANOVA) based on a pairwise means comparison by using Tukey statistical tests.

### Ethical statements: research involving plants

The research was carried out in accordance with institutional, national and international guidelines and regulations for plant experiments. The present work does not involve the use of threatened species or wild plants. Rice cultivar BRS AG (*Oryza sativa* L.) is deposited at Empresa Brasileira de Pesquisa Agropecuária (Embrapa) under the registration number 32436. Comercial cultivar BRS PAMPA (*Oryza sativa* L.) is deposited at Empresa Brasileira de Pesquisa Agropecuária under the registration number 27405. *Oryza sativa* L. (BRS AG rice) was developed and kindly donated by Embrapa Clima Temperado (Pelotas, RS, Brazil). Comercial cultivar BRS PAMPA rice was supplied by JOSAPAR (Joaquim Oliveira S.A, Rio Grande do Sul, Brazil).

### Consent to participate

All authors have given consent to participate.

### Consent to publish

All authors have approved the manuscript to be published.

## Results

### Optimization of the enzymatic hydrolysis

A study of the reaction parameters during the enzymatic hydrolysis contained in the BRS PAMPA rice was carried out. The enzymatic action time was evaluated during the starch liquefaction and saccharification. The best reaction condition obtained was used in the hydrolysis reactions of BRS PAMPA rice and BRB AG rice for fermentation reactions with the *S. cerevisiae* strain.

**Figure 1:**
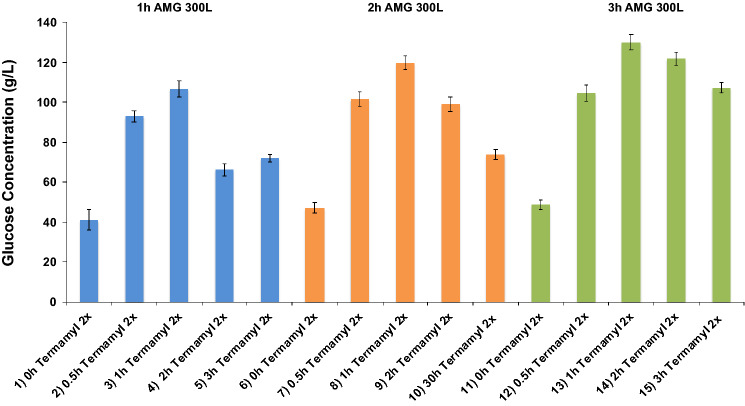
Glucose concentrations during enzymatic hydrolysis reactions with the enzymes Termamyl 2X and AMG 300L. The error bar represents the standard deviation from the essays carried out in triplicate.

During the starch liquefaction step, the action of Termamyl 2X enzyme at different reaction times was analyzed, as well as the absence of this enzyme in the process. In addition, the amyloglucosidase AMG 300L action during the saccharification step was also studied at different reaction times. Aliquots were collected and analyzed by HPLC for glucose quantification. The glucose concentration (g L^−1^) in function of the time reaction is shown in Fig. 1.

Based on the results shown in Fig. 1, it was observed that the hydrolysis 13 resulted in the highest final glucose concentration (about 130 g L^−1^). This hydrolysis was carried out with Termamyl 2X for 1-h and with AMG 300 L for 3-h. Furthermore, regardless of Termamyl 2X reaction time in the liquefaction step, a higher glucose concentration was obtained after 3 h of reaction with amyloglucosidase AMG 300 L.

Analyzing the behavior of Termamyl 2X, in the reactions without this α-amylase acting at the liquefying step, the AMG 300 L enzyme does not act efficiently in the saccharification step to obtain the glucose (Fig. 1—hydrolysis 1, 6 and 11). As the internal bonds of amylose and amylopectin were not cleaved to dextrins, the amyloglucosidase could not disrupt the α-1,4 and α-1,6-glycosidic bonds at the saccharification step, leading to a lower final concentration of glucose. Moreover, based on the bar graph, the lowest glucose concentration was obtained for hydrolysis 1, in which the AMG 300 L enzyme acted for 1 h. In the hydrolyses represented by bars 6 and 11, in which the AMG 300L reacted for 2 h and 3 h respectively, the final glucose concentration was similar, approximately 48 g L^−1^.

For the reactions in which the Termamyl 2X reacted for 30 min (Fig. 1—hydrolysis 2, 7 and 12), the lowest glucose concentration was obtained for the hydrolysis 2, in which the AMG 300 L enzyme reacted for 1 h. In the hydrolysis 7 and 12, the amyloglucosidase AMG 300L had acted for 2 h and 3 h respectively, and the final glucose concentration was similar in both cases, about 103 g L^−1^. According to Fig. 1, the reactions carried out in absence of Termamyl 2X led to the lowest glucose concentrations compared to the enzymatic reactions performed with Termamyl 2X, indicating that this enzyme plays an important role in the process related to the cleavage of α-1,4-glycosidic bonds.

The reactions with Termamyl 2X that lasted for 1 h (Fig. 1, hydrolysis 3, 8 and 13) led to the highest final glucose concentration. In hydrolysis 3, the amyloglucosidase AMG 300L has acted for 1 h, and the final glucose concentration was 107 g L^−1^. In hydrolysis 8 the AMG 300L has acted for 2 h and the final glucose concentration was about 120 g L^−1^.

The final glucose concentration obtained in hydrolysis 13 was approximately 130 g L^−1^ (Fig. 1). In this reaction, the AMG 300 L has acted for 3 h, leading to the highest yield among all evaluated reactions. This result proves that the highest glucose concentration is obtained when the time of α-amylase reaction is fixed to 1 h, and the longer is the reaction time of amyloglucosidase in the saccharification step.

The final glucose concentrations obtained in the reactions of Termamyl 2X carried out for 2 h (Fig. 1—hydrolysis 4, 9 and 14) and 3 h (Fig. 1—hydrolysis 5, 10 and 15) were much lower than the conversions found for the reactions of Termamyl 2X carried out for 1 h. Thus, it is observed that long reaction times for the liquefaction step (Termamyl 2X acting) does not lead to higher yields of glucose conversion.

**Figure 2:**
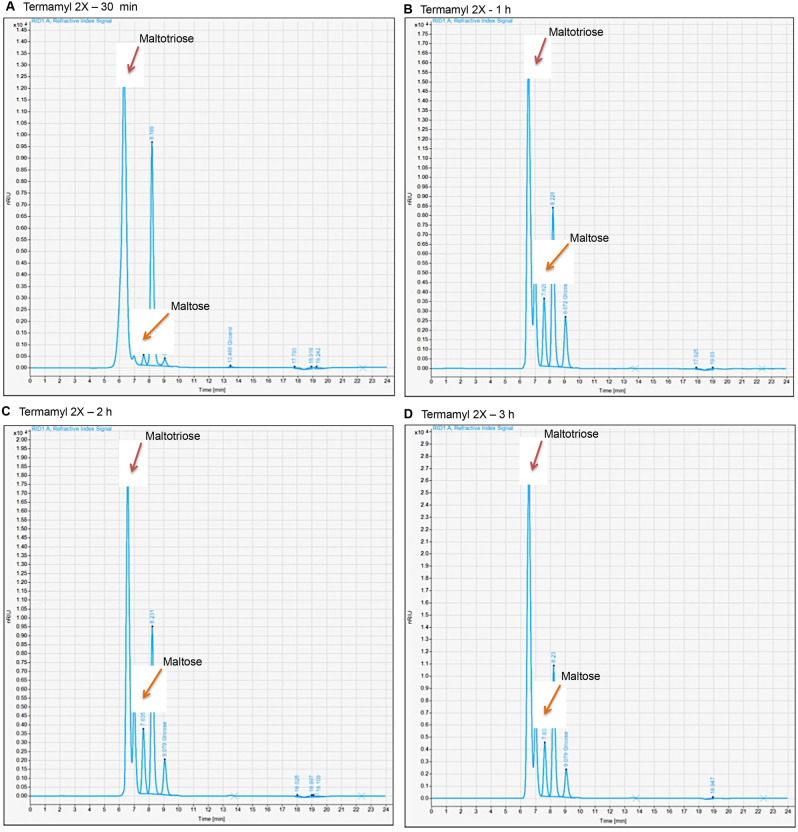
Chromatograms of the samples of the hydrolysis reactions with: **(A)** 30 min of action of Termamyl 2X; **(B)** 1 h of action of Termamyl 2X; **(C)** 2 h of action of Termamyl 2X and **(D)** 3 h of action of Termamyl 2X.

This behavior is probably due to the formation of both the maltotriose and the maltose. After 3 h of Termamyl reaction, there was possibly a greater formation of maltotriose, which possibly hampers the action of AMG 300 L during the saccharification step. It is expected that the maltotriose and the maltose are hydrolyzed at a lower rate compared to larger oligosaccharide chains^26^. Figure 2 shows the chromatograms obtained for the samples withdrawn after the action of the Termamyl 2X enzyme in different reaction times, showing a change in the behavior at the peaks with a retention time of approximately 6.5 min and 7.6 min. For 1 h of Termamyl 2X, it was observed an increase in the peak with a retention time of 7.6 min and a division or supposed overlap in the peak with a retention time of 6 min, compared to the chromatogram referring to the action of Termamyl 2X for 30 min. In particular for the reactions with Termamyl 2X acting for 2 h and 3 h, there was a significant increase in these peaks. Based on the information provided by Aminex HPLC Columns, for the HPX87H column, the compounds that are identified in these respective retention times can be maltotriose (three molecules of glucose) and maltose (two molecules of glucose)^27^.

### Fermentation of BRS PAMPA rice with different strains of *Saccharomyces cerevisiae*

**Figure 3:**
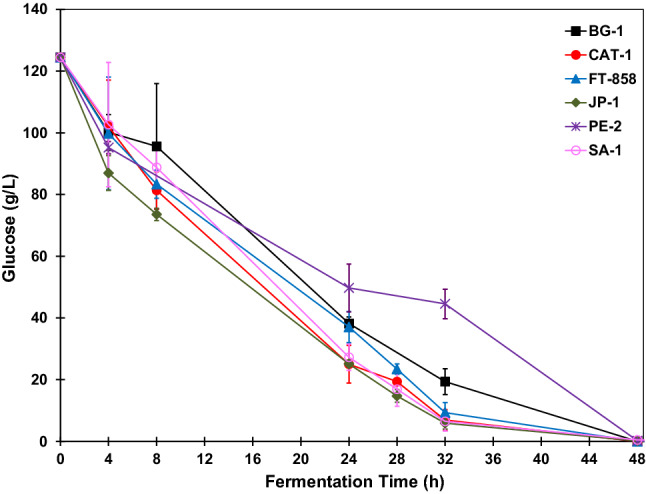
Glucose concentration profiles during BRS PAMPA rice fermentations with *S. cerevisiae* strains. The error bar represents the standard deviation from the essays carried out in triplicate.

The glucose fermentation to obtain ethanol was carried out in triplicate for each strain of *S. cerevisiae*: BG-1, CAT-1, FT-858, JP-1, PE-2, SA-1. Figure 3 shows the glucose concentrations throughout the fermentations. All glucose was consumed within 48 h after the experiment, thus the reactions were carried out for 48 h.

Based on the presented glucose concentration profiles, at the beginning of the fermentation, the glucose concentration was 124 g L^−1^. The strain JP-1 appears to be the one with the fastest kinetic reaction. This strain had a lower glucose concentration than the others, with the same reaction times, despite the values of the other strains being within the error bar of standard deviation.

The strain PE-2 had slower reaction kinetics because, after 32 h of fermentation, the glucose concentration was 45.0 ± 0.48 g L^−1^ while the other strains had a concentration around 20 g L^−1^ or below that value. At 48 h of fermentation, the strains CAT-1, BG-1, FT-858 had already consumed all the glucose. The other strains also consumed all the glucose, considering the values of the standard deviation chosen by the triplicates. The glucose concentration at 48 h of fermentation was approximately 0.17 g L^−1^ for strain JP-1, 0.20 g L^−1^ for strain PE-2 and 0.51 g L^−1^ for strain SA-1.

**Figure 4:**
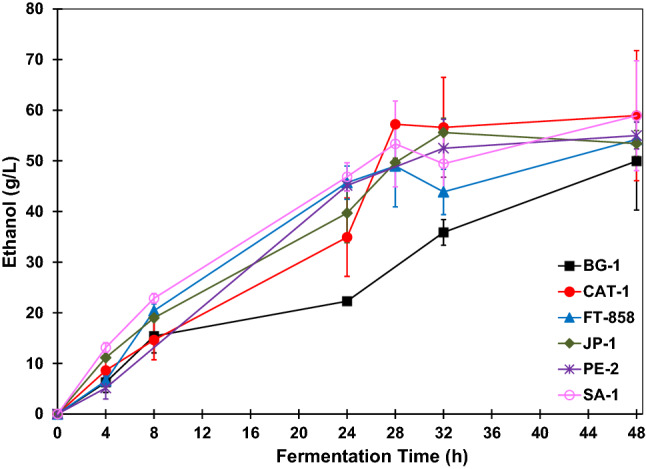
Ethanol concentration profiles during BRS PAMPA rice fermentations with *S. cerevisiae* strains. The error bar represents the standard deviation from the essays carried out in triplicate.

**Figure 5:**
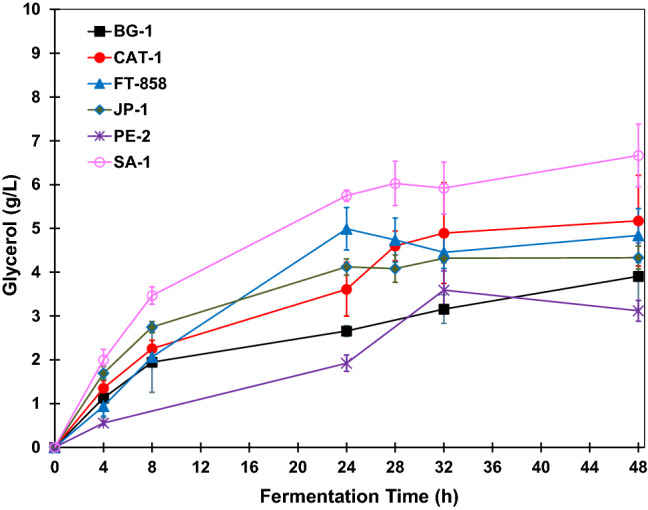
Glycerol concentration profiles during BRS PAMPA rice fermentations with *S. cerevisiae* strains. The error bar represents the standard deviation from the essays carried out in triplicate.

The ethanol concentration profiles are shown in Fig. 4. It was observed that after 4 h of fermentation, the strain SA-1 produced the largest amount of ethanol, 13 g L^−1^. In contrast, PE-2 led to the lowest ethanol concentration for the same time of reaction, approximately 2 g L^−1^. The ethanol concentration profile of BG-1 shows that this strain had more difficulty in converting glucose into ethanol compared with the other strains since the ethanol concentration was the lowest. After 48 h of fermentation, the ethanol final concentration for BG-1 was about 50 g L^−1^. The strains that produced a greater amount of ethanol were CAT-1 and SA-1, with a final concentration of approximately 59 g L^−1^. The strains FT-858 and PE-2 produced about 54 g L^−1^ and 55 g L^−1^ ethanol after 48 h of fermentation, respectively. The strain JP-1 produced 53 g L^−1^ of ethanol after 48 h of fermentation. Besides ethanol, which was the product of interest, all strains produced glycerol as a co-product during the fermentations, as shown in Fig. 5.

The glycerol concentration during the fermentative reactions shows a similarity for all strains of *S. cerevisiae*. However, throughout the fermentation, strain SA-1 had produced higher concentrations of glycerol. In this way, this strain was in conditions of greater stress compared to the others since the reaction conditions are determinants for the cellular metabolism of the strains.

There exist many stressful yeast conditions that provide a lower yield and higher formation of co-product. Some of them are fermentation temperature that usually varies between 32 and 35 °C; ethanol concentration that reaches 8%–11% (v/v) towards the end of each fermentation cycle and bacterial contamination.

In addition to osmotic stress, substrate concentration, agitation rate, time of fermentation reaction and fermentative medium pH are factors that can cause yeast stress, leading to a higher production of co-products. At the end of fermentation, which occurred after 48 h, the glycerol concentration produced by strain SA-1 was 6.7 g L^−1^. The strain, which produced the second-highest glycerol concentration, was CAT-1, with approximately 5.2 g L^−1^. In contrast, strain PE-2 was the one that produced the lowest amount of glycerol. After 48 h the glycerol concentration was 3.1 g L^−1^ for this strain. The strains FT-858, JP-1, BG-1 yielded 4.8 g L^−1^, 4.3 g L^−1^ and 3.9 g L^−1^, respectively.

After analyzing the concentration profiles for glucose consumption, ethanol production and glycerol, we calculated the fermentation yield, glucose (substrate) conversion factors in ethanol (Y_E/S_)_,_ glucose conversion factors in glycerol (Y_G/S_), productivity and rates of substrate consumption. The results obtained are shown in Table 1.

**Table 1:** BRS PAMPA rice fermentation parameters with different strains of *Saccharomyces cerevisiae* (N.B. standard deviation was determined from the essays carried out in triplicate).

	BG-1	CAT-1	FT-858	JP-1	PE-2	SA-1
Ethanol (g L^−1^)	49.96 ± 9.67	58.93 ± 12.85	54.22 ± 4.12	53.43 ± 2.57	55.01 ± 2.67	58.92 ± 10.83
Glycerol (g L^−1^)	3.91 ± 0.76	5.17 ± 1.04	4.84 ± 0.62	4.33 ± 0.27	3.12 ± 0.24	6.67 ± 0.72
Yield (%)	78.56	92.68	85.27	84.14	86.64	93.04
Y_E/S_	0.40	0.47	0.44	0.43	0.44	0.48
Y_G/S_	0.03	0.04	0.04	0.04	0.03	0.05
Productivity (g_ethanol_ L^−1^ h^−1^)	1.04	1.23	1.13	1.11	1.15	1.23
Substrate consumption rates (g_glucose_ L^−1^ h^−1^)	2.59	2.59	2.59	2.59	2.59	2.58
Residual glucose (g L^−1^)	0.00	0.00	0.00	0.17 ± 0.30	0.20 ± 0.35	0.51 ± 0.23

Based on the parameters obtained and shown in Table 1, the maximum yield was obtained by using strain SA-1 in fermentation reaction. This strain had a 93.0% yield, and 58.92 ± 10.83 g L^−1^ as final ethanol concentration. However, after 48 h of fermentation, the substrate had not been fully consumed, leaving 0.51 ± 0.23 g L^−1^ as residual glucose concentration. The strain CAT-1 presented the second-highest yield with 92.7% and had consumed at all the glucose. The final ethanol concentration obtained was 58.93 ± 12.85 g L^−1^.

Based on ethanol production curves for the fermentations carried out with these two strains (SA-1 and CAT-1) presented in Fig. 4, the final ethanol concentration can be considered statistically equivalent. However, the glycerol concentration was lower for CAT-1, with a concentration of 5.17 ± 1.04 g L^−1^, while for SA-1 the glycerol concentration was 6.67 ± 0.72 g L^−1^. Thus, the yield of the SA-1 strain was slightly higher and formed more glycerol as a fermentation co-product than CAT-1.

The strains FT-858, PE-2 and JP-1 showed similar fermentation parameters. The yield of FT-858 was 85.3% and the final ethanol concentration was 54.22 ± 4.12 g L^−1^. The yield of PE-2 was 86.6% and the final ethanol concentration was 55.0 ± 2.67 g L^−1^. The final glycerol concentration was higher for FT-858, 4.84 ± 0.62 g L^−1^ while the glycerol concentration for PE-2 was 3.12 ± 0.24 g L^−1^. The total substrate consumption occurred for the strain FT-858. Considering that the residual glucose for PE-2 was 0.20 ± 0.35 g L^−1^, it can be concluded that this strain also consumed all the glucose during the fermentation. The process productivity and the rate of substrate consumption were the same for these two strains, 1.12 g L^−1^ and 2.59 g L^−1^, respectively. Strain JP-1 had an 84.1% as fermentation yield. The final ethanol concentration was 53.43 ± 2.57 g L^−1^and the final glycerol concentration was 4.33 ± 0.27 g L^−1^. As with PE-2, it can be assumed that this strain consumed all the substrate since the residual glucose concentration was 0.17 ± 0.30 g L^−1^.

The fermentation carried with strain BG-1 showed the lowest yield, 78.6%, despite all the glucose being consumed and high concentrations of glycerol, 3.91 ± 0.76 g L^−1^, were not formed. As shown in Fig. 4, throughout the fermentation this strain produced lower concentrations of ethanol compared to the other strains. Therefore, this strain had obtained the lowest productivity value of 1.04 g_ethanol_·L^−1^·h^−1^.

The BRS PAMPA rice fermentations that presented the highest yield and highest ethanol concentration were those carried out with strains CAT-1 and SA-1. However, SA-1 had not consumed the substrate at all and had led to the highest glycerol concentration, while strain CAT-1 consumed the glucose at all and had led to a lower concentration of glycerol as a co-product. High yield is also observed in industrial-scale fermentations when strain CAT-1 is used^24^.

Each *S. cerevisiae* commercial strain has specific characteristics that are considered when choosing the fermentation process. Brazilian distilleries have many strains on the market to use in industrial fermentation processes, however, the strains most used at fermentations to obtain ethanol are CAT-1 and PE-2. These two strains represent 70% of the Brazilian market for fuel ethanol production^23^. The strain CAT-1 was isolated from the Catanduva and the strain PE-2 from Pedra plants^7^. These strains are more adapted to industrial conditions and provide higher ethanol yield and lower amount of residual sugar. In addition, they produce little foam during fermentation and do not flocculate^28^.

**Figure 6:**
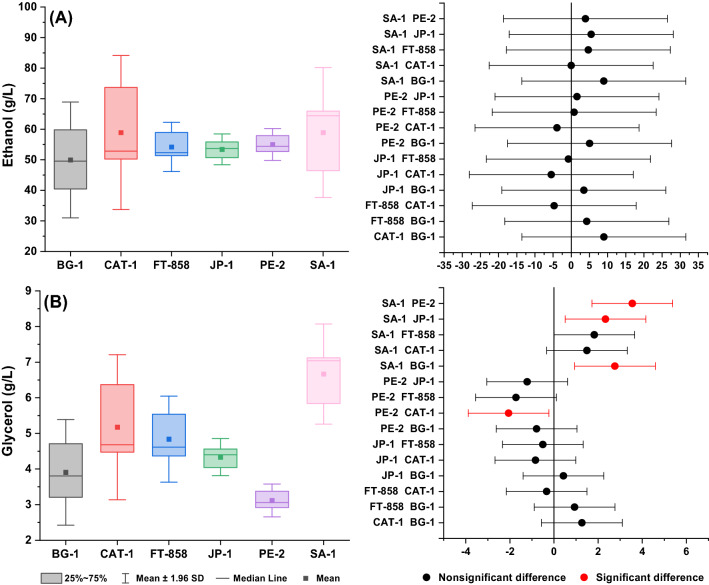
Box charts and means comparison using Tukey mean-difference plot for **(A)** ethanol production and **(B)** glycerol production related to BRS PAMPA rice fermentations with *S. cerevisiae* strains.

It is generally agreed that statistical evaluation of the experimental data provides fundamental insight into the fermentation behavior as well as the comparative performance of *S. cerevisiae* commercial strain. It is important to bear in mind that from a statistical point of view the ability of all strains, evaluated in the present work, to convert BRS PAMPA rice into ethanol can be considered equivalent, as depicted in Fig. 6A. Although CAT-1 and SA-1 exhibit the highest average value for ethanol concentration (approximately 59 g L^−1^) compared to the other strains, based on the ANOVA statistical test carried out with a significance level of 0.05, the mean values of ethanol concentration determined for all evaluated strains are not significantly different. On the other hand, when the concentration of produced glycerol is considered, ANOVA results come back with a different outcome (Fig. 6B), showing that the mean value obtained of the glycerol production by strain SA-1 t (with the highest mean value, approximately 7 g L^−1^) is statistically different from the one determined for PE-2, JP-1 and BG-1, and PE-2 is significantly different from CAT-1. Based on glucose consumption and glycerol formation (Table 1), it is reasonable to assume the CAT-1 may be considered as the best strain for the ethanol production from high-amylopectin BRS PAMPA rice.

### Fermentation of BRS AG rice with different strains of *Saccharomyces cerevisiae*

**Figure 7:**
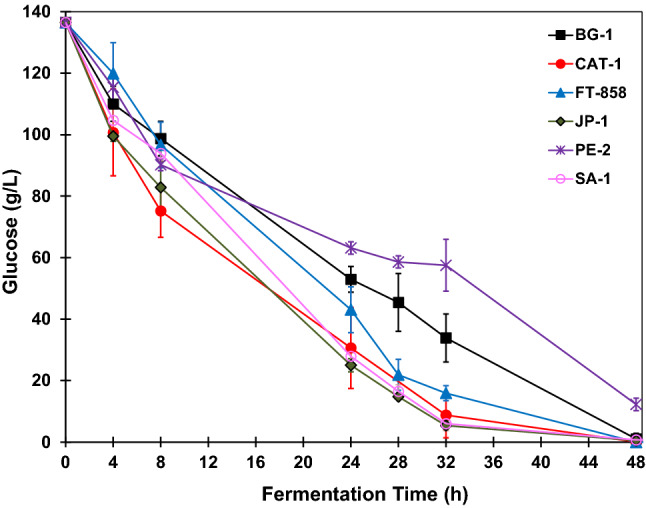
Glucose concentration profiles during BRS AG rice fermentations with *S. cerevisiae* strains. The error bar represents the standard deviation from the essays carried out in triplicate.

The glucose fermentation with BRS AG rice to obtain ethanol was carried out in triplicate for each strain of *S. cerevisiae*: BG-1, CAT-1, FT-858, JP-1, PE-2, SA-1. Figure 7 shows the glucose concentrations throughout the fermentations. All glucose was consumed within 48 h after the experiment, thus the reactions were carried out for 48 h.

Based on the presented glucose concentration profiles (Fig. 7), at the beginning of the fermentation, the glucose concentration was 136.5 g L^−1^. The strains CAT-1, JP-1 and SA-1 presented faster reaction kinetics compared to the other strains. These strains along with FT-858 had lower glucose concentrations with the same reaction times. In contrast, PE-2 was the slowest reaction kinetics with the lowest glucose consumption. After 48 h of fermentation, the glucose concentration in the fermentation medium was about 12.26 ± 2.02 g L^−1^ for this strain. The strain BG-1 also had slower reaction kinetics in comparison to the strains CAT-1, FT-858, JP-1 and SA-1 (Fig. 7), however considering the residual glucose concentration of 1.03 ± 1.79 g L^−1^ after 48 h of reaction, it is reasonable to assume that substrate was significantly consumed. The strain FT-858 had already consumed all the glucose after 48 h of fermentation. The strains JP-1 and SA-1 had not consumed the glucose at all, leaving residual glucose in the fermentative medium with a concentration near zero. The glucose concentration with 48 h of fermentation was 0.53 ± 0.09 g L^−1^ for strain JP-1 and 0.72 ± 0.21 g L^−1^ for strain SA-1.

**Figure 8:**
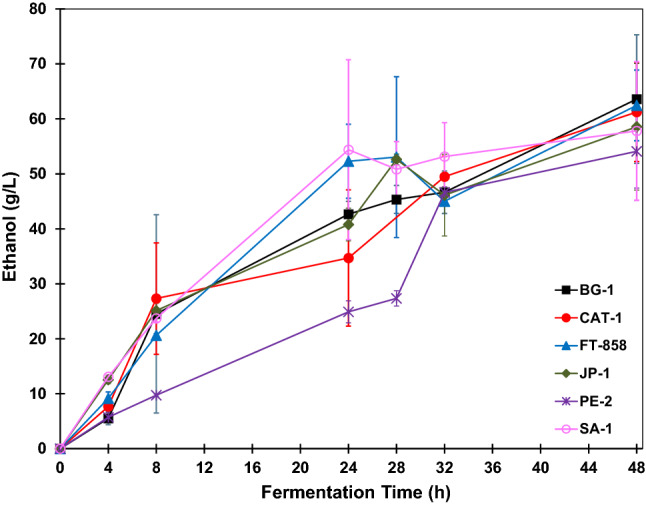
Ethanol concentration profiles during BRS AG rice fermentations with *S. cerevisiae* strains. The error bar represents the standard deviation from the essays carried out in triplicate.

The ethanol concentration profiles during the fermentations are shown in Fig. 8. It was observed that at 4 h of fermentation the strains that produced the highest amounts of ethanol were JP-1, with a concentration of 12.56 ± 0.74 g L^−1^, and SA-1, with a concentration of 13.09 ± 0.32 g L^−1^. On the other hand, BG-1 and PE-2 were the strains that had the lowest ethanol concentration for the same reaction time, 5.54 ± 1.17 g L^−1^ and 5.73 ± 0.83 g L^−1^, respectively. The ethanol concentration profile of PE-2 demonstrated a greater difficulty of this strain in the conversion of glucose into ethanol, compared with the other strains. This is because strain PE-2 obtained the lowest ethanol concentrations during the fermentation and had not consumed all the glucose present in the fermentative medium. After 48 h of fermentation, the final ethanol concentration for this strain was about 54.10 ± 6.67 g L^−1^. The strains that produced a greater amount of ethanol were FT-858 with a concentration of 62.45 ± 6.43 g L^−1^ and CAT-1, with a concentration of 61.25 ± 9.02 g L^−1^. The strain JP-1 produced 58.56 ± 11.52 g L^−1^ of ethanol after 48 h of fermentation. The final ethanol concentration for strain BG-1 was 54.67 ± 4.36 g L^−1^, and for strain SA-1 was 57.80 ± 12.61 g L^−1^.

**Figure 9:**
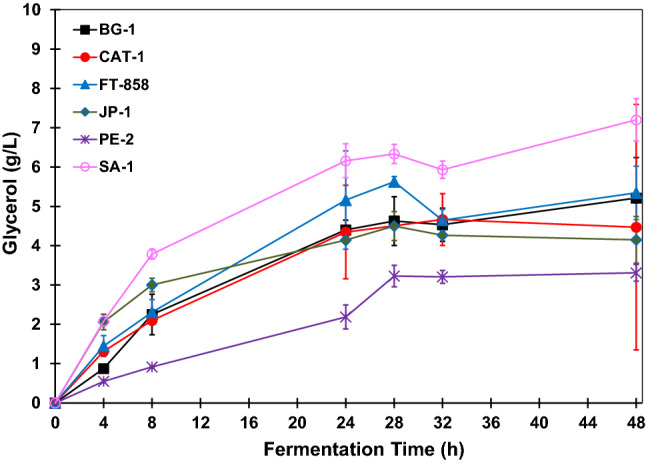
Glycerol concentration profiles during BRS AG rice fermentations with *S. cerevisiae* strains. The error bar represents the standard deviation from the essays carried out in triplicate.

The glycerol concentration profiles obtained as co-product in all fermentations performed with BRS AG rice is shown in Fig. 9. As with fermentations carried out with BRS PAMPA rice, during the whole fermentation process, the strain SA-1 produced higher concentrations of glycerol, demonstrating that this strain was in conditions of greater stress compared to the others. This condition may be related to the high osmotic pressure of the fermentation medium, as well as adverse conditions such as acid pH, high temperature, high substrate concentration and even the presence of contaminants in the medium fermentative. These factors corroborate in the production of fermentation co-products, such as glycerol^18,29^.

The strain that led to the highest glycerol concentration after 48 h of fermentation was SA-1, with 7.20 ± 0.54 g L^−1^. The strain that produced the second-highest glycerol concentration was CAT-1, with 5.82 ± 0.80 g L^−1^. In contrast, strain PE-2 was the that produced the lowest amount of glycerol. After 48 h the glycerol concentration was 3.31 ± 0.21 g L^−1^ for this strain. The strains FT-858, BG-1 and JP-1 produced 5.34 ± 0.68 g L^−1^, 5.21 ± 1.03 g L^−1^ and 4.15 ± 0.59 g L^−1^, respectively.

Based on the results, a similarity in the curves of glycerol production is observed in the fermentations with the BRS PAMPA rice and BRS AG rice. For both cases, the strains that led to a higher formation of glycerol, as a co-product, was SA-1, followed by CAT-1 and FT-858. The only difference for the fermentations of these two types of rice in relation to the final glycerol concentration was that in the BRS PAMPA rice case, the strain that led to the fourth-largest concentration of this co-product was JP-1, and in the case of BRS AG rice, was strain BG-1. The lowest glycerol concentration obtained for both was for fermentation carried out with the strain PE-2.

After 48 h of reaction, the glucose consumption, ethanol and glycerol production were analyzed for the fermentation of BRS AG rice. The fermentations yield was calculated, glucose (substrate) conversion factors in ethanol (Y_E/S_), glucose conversion factors in glycerol (Y_G/S_), productivity and consumption substrate rates. The results obtained are shown in Table 2.

**Table 2:** BRS AG rice fermentation with different strains of *Saccharomyces cerevisiae* parameters (N.B. standard deviation was determined from the essays carried out in triplicate).

	BG-1	CAT-1	FT-858	JP-1	PE-2	SA-1
Ethanol (g L^−1^)	54.67 ± 4.36	61.25 ± 9.02	62.45 ± 6.43	58.56 ± 11.52	54.10 ± 6.67	57.80 ± 12.61
Glycerol (g L^−1^)	5.21 ± 1.03	5.82 ± 0.80	5.34 ± 0.68	4.15 ± 0.59	3.31 ± 0.21	7.20 ± 0.54
Yield (%)	78.99	87.87	89.55	84.30	85.22	83.32
Y_E/S_	0.40	0.45	0.46	0.43	0.44	0.43
Y_G/S_	0.04	0.04	0.04	0.03	0.03	0.05
Productivity (g_ethanol_ L^−1^ h^−1^)	1.14	1.28	1.30	1.22	1.13	1.20
Substrate consumption rates (g_glucose_ L^−1^ h^−1^)	2.82	2.84	2.84	2.83	2.59	2.83
Residual glucose (g L^−1^)	1.03 ± 1.79	0.06 ± 0.11	0.00	0.53 ± 0.09	12.26 ± 2.02	0.72 ± 0.21

Based on the parameters obtained for the fermentations with different strains of *S. cerevisiae* presented in Table 2, the strain that presented the highest yield was FT-858, with 89.6%. The strain CAT-1 presented the second-highest yield, with 87.9% and had consumed all the glucose. In addition, productivity was similar for these two strains, 1.30 g_ethanol_ L^−1^ h^−1^ for strain FT-858 and 1.28 g_ethanol_ L^−1^ h^−1^ for CAT-1, with equal rate of substrate consumption of 2.84 g_glucose_ L^−1^ h^−1^.

Compared to the fermentation carried out with strain BG-1, the parameters obtained for the fermentation with JP-1 were better. The yield obtained for strain JP-1 was 84.3% and for strain BG-1 was 79.0%. The process productivity was lower for BG-1 (1.14 g_ethanol_ L^−1^ h^−1^) and JP-1 (1.22 g_ethanol_ L^−1^ h^−1^). The rate of substrate consumption was similar, 2.82 g_glucose_ L^−1^ h^−1^ for strain BG-1 and 2.83 g_glucose_ L^−1^ h^−1^ for strain JP-1. The strain PE-2 showed 85.2% of yield with the lowest productivity value (1.13 g_ethanol_ L^−1^ h^−1^) and a rate of substrate consumption of 2.59 g_glucose_ L^−1^ h^−1^. The fermentation carried out with strain SA-1 showed a 83.3% yield, exhibiting a substrate consumption rate equal to 2.83 g_glucose_ L^−1^ h^−1^ and productivity of 1.20 g_ethanol_ L^−1^ h^−1^.

Thus, for both fermentations carried out with BRS PAMPA and BRS AG rice, the strain CAT-1 led to the second-highest yield and final ethanol concentration. Industrially, this strain is the most used in fermentations of sugarcane juice. One reason is the aluminum tolerance of cane juice. The strain FT-858 is also tolerant to aluminum and is used to reduce the problems caused by this metal during fermentation^23,28^.

The reaction conditions were determinant in the final concentration of ethanol and glycerol production. Stressful conditions for the yeast provide a lower yield and higher formation of this co-product. Higher amounts of glycerol are obtained when the yeast is under osmotic stress since in this condition the yeast loses water and initiates the synthesis of glycerol to protect the cells from dehydration and protect them from the effects arising from the stress condition^24^. In addition to osmotic stress, substrate concentration, agitation rate, presence of contaminants, fermentation time, pH and temperature of the fermentative medium are factors that can cause yeast stress and lead to a higher formation of co-products and lower yield of the product of interest, ethanol^17,18,23^.

In fermentations carried out for 48 h with the different strains of *S. cerevisiae*, both rice cultivars led to high ethanol yields. However, BRS AG rice led to yields close to 90%, with four strains (CAT-1 and FT-858) and yields between 80 and 85% (BG-1, JP-1, PE-2 and SA-1). On the other hand, the yields obtained for BRS PAMPA rice were above 90% for CAT-1 and SA-1 strains. The yield was around 85% for three strains (FT-858, JP-1, PE-2). The lowest yield was obtained for the fermentation using BRS PAMPA rice and the BG-1 strain and was 78.6%. With these results, it can be said that the rice developed by Embrapa Clima Temperado proved to be an excellent alternative for ethanol production by fermentation with different *S. cerevisiae* strains compared to conventional rice BRS PAMPA.

**Figure 10:**
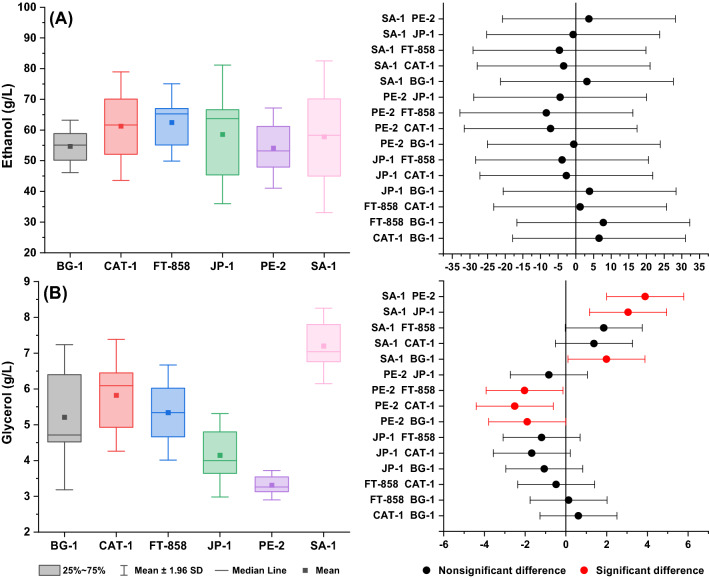
Box charts and means comparison using Tukey mean-difference plot for **(A)** ethanol production and **(B)** glycerol production related to BRS AG rice fermentations with *S. cerevisiae* strains.

Although the profiles of ethanol and glycerol production seem to be relatively different throughout the fermentation time (Figs. 8 and 9), it also seems that the final ethanol concentration was the statistically same. For this reason, a statistical analysis considering the mean values at the end of the fermentation (48 h) was performed to give a piece of comparative information about the ability of the strains to produce ethanol and glycerol, as shown in Fig. 10. According to ANOVA statistical test, mean values of the ethanol concentration at the end of the BRS AG rice fermentations carried out with all strains cannot be considered different, as illustrated in Fig. 10A. However, when the final glycerol concentration at 48 h fermentation is considered in the statistical analysis, the comparative result from the Tukey test showed that performance of SA-1, which is the strain that led to the highest mean value of glycerol concentration (7.2 g L^−1^) is statistically different from the other strains PE-2, JP-1 and BG-1, while the strain led to the lowest glycerol concentration (3.3 g L^−1^), PE-2 is significantly different from FT-858, CAT-1 and BG-1. It is worth mentioning that both CAT-1 and FT-858 have very similar mean values of ethanol concentration (61 g L^−1^ and 62 g L^−1^, respectively) and glycerol concentration (5.8 g L^−1^ and 5.3 g L^−1^, respectively), which is also not statistically different according to ANOVA assessment (Fig. 10). Based on this analysis, CAT-1 and FT-858 might be considered as the most preferred strains to produce ethanol from high-amylopectin BRS AG rice.

## Conclusions

The new rice cultivar BRS AG proved to be an excellent biomass to produce ethanol, observing that for all strains of *Saccharomyces cerevisiae* studied, the ethanol yield was greater than or equal to 79.0%, presenting similar performance when compared to the commercial cultivar BRS PAMPA that presented yields for ethanol higher than 78.6%.

The study of different commercial *Saccharomyces cerevisiae* strains determined that the highest yields and higher ethanol concentrations were obtained for strain SA-1 (93% and ethanol concentration of 58.92 ± 10.83 g L^−1^) and for the strain CAT-1 (92.7% and ethanol concentration of 58.93 ± 12.85 g L^−1^), for fermentations with the BRS PAMPA rice. For the fermentations with BRS AG rice, the highest yields and higher ethanol concentrations were obtained with the strain FT-858 (89.6% and ethanol concentration of 62.45 ± 6.43 g L^−1^), and CAT-1 (87.9% and ethanol concentration of 61.25 ± 9.02 g L^−1^). The CAT-1 is one of the most used strains in the fermentation of sugarcane industrially and with the results obtained through this study, it can be concluded that this strain also performs satisfactorily in the fermentations with rice.

To avoid competition with the food industry, Embrapa Clima Temperado has developed a genetically modified rice cultivar to be used in the production of ethanol. This cultivar is not recommended for human consumption and its planting is in lowland fields, characterized by presenting hydromorphic, poorly drained soils. This demonstrates that this cultivar does not compete with the planted area of other cultures such as soybean, maize, and sorghum, even the conventional rice.

## Data Availability

The authors confirm that the data and materials supporting the findings of this article are available within the article and from the corresponding author, [SBG], upon reasonable request.
